# Mechanical Performance and Energy Absorption of Ti6Al4V I-WP Lattice Metamaterials Manufactured via Selective Laser Melting

**DOI:** 10.3390/ma18194626

**Published:** 2025-10-07

**Authors:** Le Yu, Xiong Xiao, Xianyong Zhu, Jiaan Liu, Guangzhi Sun, Yanheng Xu, Song Yang, Cheng Jiang, Dongni Geng

**Affiliations:** 1 School of Mechanical and Aerospace Engineering, Jilin University, Changchun 130022, China; yule23@mails.jlu.edu.cn (L.Y.); xiaoxiong20@mails.jlu.edu.cn (X.X.); xuyanhengxyh@163.com (Y.X.); yangsongjlu@jlu.edu.cn (S.Y.); jiangcheng@jlu.edu.cn (C.J.); gengdongni2005@jlu.edu.cn (D.G.); 2 College of Materials Science and Engineering, Jilin University, Changchun 130022, China; liuja@jlu.edu.cn (J.L.); sungz23@mails.jlu.edu.cn (G.S.)

**Keywords:** selective laser melting, mechanical properties, metamaterials, energy absorption

## Abstract

Metamaterial lattice structures based on a Triply Periodic Minimal Surface (TPMS) structure have attracted much attention due to their excellent mechanical properties and energy absorption capabilities. In this study, a novel TPMS lattice metamaterial structure (IWP-X) is designed to enhance the axial mechanical properties by fusing an X-shaped plate with an IWP surface structure. A selective laser melting (SLM) machine was utilized to print the designed lattice structures with Ti6Al4V powder. The thickness of the plate and the density of the IWP are varied to explore the responsivity of the mechanical and energy absorption properties with the volume ratio of IWP-X. The finite element simulation analysis is used to effectively predict the stress distribution and fracture site of each structure in the compression test. The results show that the IWP-X structure obtains the ultimate compressive strength of 122.06% improvement, and the energy absorption of 282.03% improvement. The specific energy absorption (SEA) reaches its maximum value in the plate-to-IWP volume ratio of 0.7 to 0.8.

## 1. Introduction

Lattice materials are not only lightweight and high-strength, but also possess a lot of high-performance complex structures, which have high applications in the fields of healthcare, transportation, aerospace, etc. [1,2,3,4,5,6]. However, the difference in properties between different lattice structures is also relatively obvious, and their structure has a decisive role in material properties. Additive manufacturing technology through powder layup laser melting enables the application of high-performance lattice structures that previously could not be fabricated by conventional means [7,8,9,10,11,12]. How to create a lattice structure with superior performance has become a hot research topic in recent years.

Common lattice structures include truss lattices such as the body-centered cubic structure (BCC) [13] and face-centered cubic structure (FCC) [14], plate lattices such as thin-walled crystal plate lattices (CPLs) [15], and the Triply Periodic Minimal Surface (TPMS) structure [16,17,18,19]. With the increasing research on lattice metamaterials, the creativity of TPMS structures with smooth inner walls generated by implicit functions is superior to that of other lattice structures. Guan et al. [20] designed a dual-material P-surface TPMS metamaterial to optimize structural performance and improve structural load-bearing capacity. Zhao et al. [21] propose a design approach based on TPMS for interpenetrating lattice structures to develop a multiscale optimization framework.

Fusing different structures is now a popular way to study lattice metamaterials. Naji et al. [22] presented a novel hybrid plate–TPMS lattice that increased 171% in modulus, 125% in yield strength, 118% in plateau stress, and 117% in SEA. Zhang et al. [23] incorporated shell-based and plate-based lattices to design a mechanically stable metamaterial structure with significantly enhanced mechanical properties. However, the method of reinforcing the structure with I-Wrapped Package surface (I-WP) fusion plates is less explored.

In this study, we combined the vertical X-shaped plate with the I-WP, and designed the IWP-X Plate structure (IWP-X) with different porosities and plate thickness structures to study the effect of the plates on the strength and energy absorption properties of the I-WP structure. The designed structures were fabricated by the selective laser melting (SLM) technique using Ti6Al4V powder. Finite element simulation (FEA) tests and compression tests were conducted to investigate the mechanical properties of the new metamaterial structure and to fit the law of the influence of volume ratio on the structure.

## 2. Materials and Methods

### 2.1. Structural Design

In this study, the X-shaped plate reinforcement structure is integrated with the I-WP surface structure. The formula for generating the I-WP surface structure is [24]

(1) $$\phi_{IWP}\left(x,y,z\right)=\cos\left(\omega x\right)\cos\left(\omega y\right)+\cos\left(\omega y\right)\cos\left(\omega z\right)+\cos\left(\omega z\right)\cos\left(\omega x\right)-0.5\left[\cos\left(2\omega x\right)+\cos\left(2\omega y\right)+\cos\left(2\omega z\right)\right]+c$$

where *x*, *y*, and *z* represent spatial coordinates, *ω* = 2*πn*/*l*, *n* is the number of the unit cell, *l* is the length of the unit cell, and *c* is the boundary constant controlling porosity.

**Figure 1:**
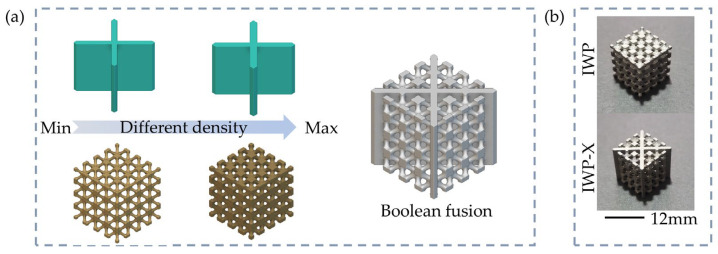
(**a**) Boolean fusion of IWP-X lattice structures; (**b**) the SLM specimens for IWP and IWP-X lattice structures.

A total of nine new structures were designed, including three structures with the same density (IWP45-0.7, IWP45-1, and IWP45-1.3), three structures with the same thickness of plate (IWP40-1, IWP50-1, and IWP55-1), and three structures with the same volume of I-WP surface structure (IWP40-0.7, IWP50-1.25, and IWP55-1.5). These structures were compared with the original I-WP structure. As shown in Figure 1b, the I-WP surface structure is generated by MATLAB R2023b software, and the plate structure is generated by SolidWorks 2020 software. We control the volume of IWP by turning the boundary constant *c*, and the volume of the structures is measured by the Materialise Magics 24.0 software. In this paper, relative density is related to volume:

(2) $$\rho^{*}=\frac{V_{IWP}}{{l_{IWP}}^{3}}$$

where $\rho^{*}$ is the relative density of the structure, $V_{IWP}$ is the volume of the IWP structure, and $l_{IWP}=12\;mm$ is the length of the structure.

The number of unit cells of the designed structures is 4 × 4 × 4. Each unit cell is a 3 mm cube. Each structure was designed with a 1 mm thick base plate at the bottom for cutting after SLM printing. The pictures for the specimens are shown in Figure 1b, and the design parameters of the lattice structures are summarized in Table 1. The formula for the volume of the plate is

(3) $$V_{t}={l_{IWP}}^{3}-l_{IWP}(l_{IWP}-\sqrt{2}t)2$$

where $V_{t}$ is the volume of the plates, and t is the thickness of the plate. The ratio of the volume is *V_t_*/*V_IWP_*, and the *V_IWP_* is the volume of the original IWP structure before fusion.

### 2.2. Sample Preparation

**Figure 2:**
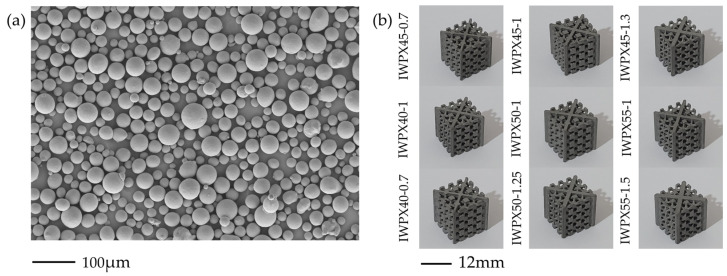
(**a**) SEM of the Ti6Al4V powder; (**b**) as-built samples.

All structures are manufactured by a high-precision selective laser melting machine (YLMs-1, China). As shown in Figure 2a, the powder was Ti6Al4V powder with a particle size of 15–53 µm, used after ultrasonic vibration sieving. The specific particle size distribution of the powder was measured by a laser particle size analyzer (LS-POP9, China), and the results were as follows: D10 = 18.48 µm, D50 = 24.22 µm, and D90 = 49.76 µm. The parameters of the SLM machine are listed in Table 2, and the scan strategy is meander scanning with no rotation between layers. The chemical composition of the Ti6Al4V powder is shown in Table 3, which is consistent with the other literature [25,26]. Mechanical properties of titanium alloy powder materials from powder suppliers are shown in Table 4, which are similar to the properties of Ti6Al4V materials in the other literature [27]. The whole fabrication process was carried out in high-purity argon gas to avoid powder explosion. All specimens were cut off with wire electrical discharge after printing, and then the residual substrate powder inside the cytosol was cleaned with ultrasonic waves to avoid blocking the air holes and affecting the experimental results. No heat treatment was conducted after printing. As shown in Figure 2b, the sample appearance is consistent with the design.

### 2.3. Finite Element Simulation

To predict the mechanical properties and fracture sites of the samples, finite element simulation experiments were performed using the ABAQUS/Explicit 2020 nonlinear solver platform. The meshing of the grid structure was performed using nTopology software with a mesh size of 0.2 mm and 10-node tetrahedral grid cells of type C3D10. The number of nodes and elements is shown in Table 5. The simulation module uses explicit dynamics, assuming that the material is isotropic, and the plastic deformation parameters are obtained from the real tensile curves of the tensile experimental structures. Related papers are set up using the same method [28,29,30].

**Figure 3:**
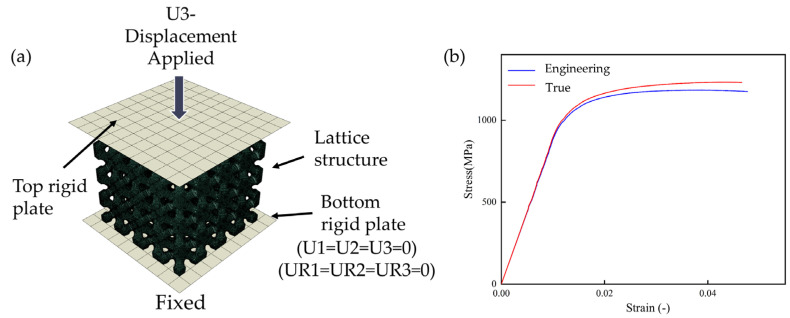
(**a**) Numerical models of the compressive test for a lattice structure; (**b**) stress–strain curves of the tensile sample for model input.

The modulus of elasticity (104 GPa) of the Ti6Al4V material was obtained from tensile tests on tensile specimens fabricated, and the tensile parts used for the tests were produced by the same printer using the same powder and printing parameters. Poisson’s ratio was set to 0.33 as derived from the literature [31], and the density was set to 4.45 g/cm^3^ as provided by the powder supplier. The tensile structure is shown in Figure 3b. The parameters of the model of the simulation tests were derived from the results of the stress–strain curves of the tensile tests.

As shown in Figure 3a, the upper and lower rigid plate properties are placed on the upper and lower sides of the compressed structure using discrete stiffeners, and the contact with the compressed structure is set as a general contact with a friction coefficient of 0.2. The FEA adopts a smoothing analysis method to ensure a smooth transition at the beginning of the loading and smooth convergence of the computational process. The upper rigid plate is set to be compressed downward with a speed of 2 mm/min in the Z-axis while being restricted by rotation and translation in other directions. The lower rigid plate is set to be a fixed base, and the reference point is set to be at the center of the upper compression plate.

### 2.4. Mechanical Performance Test

The quasi-static compression and tensile test processes are completed on the Instron-5869 universal testing machine. During the compression test, the sample is placed in the middle of two steel plates. The top plate moves downward at a speed of 2 mm/min, and the compression process is filmed with a high-speed video camera. The yield strength of structures is obtained at 0.2% strain.

The energy absorption capacity is a key factor in characterizing the energy absorption properties of lattice structures and can be calculated by integrating the area under the engineering stress–strain curve and plotting the energy absorption curves. The energy absorption per unit volume (*W*) during compression can be calculated by [32]

(4) $$W=\int_{0}^{\varepsilon }\sigma \left(\varepsilon \right)\mathrm{d}\varepsilon$$

where *W* is the energy absorption per unit volume, σ is the compressive stress, and ε is the strain. The final energy absorption value of the structure is obtained during integration into the fracture strain.

In addition, the specific energy absorption (SEA) is used to evaluate the energy absorption capacity of structures with different relative densities, which can be expressed by the following relationship [33]:

(5) $$SEA=\frac{W}{\rho }$$

where *W* is the energy absorption per unit volume, and ρ is the density of structures.

Three samples of each structure were printed (30 specimens in total), and the results showed consistency. The stress–strain curves were derived from one of the sets of results, and the mechanical properties were obtained by the mean value of the data. In this study, the stress value is set as the compressive force divided by the initial cross-sectional area perpendicular to the loading direction (sample length × sample width), and the strain value is the compressive displacement divided by the sample height. This way of taking the values of stresses is also used in the other literature [34,35,36].

### 2.5. Characterization Test

The mass and volume of the specimens were measured. The formula for calculating relative density $\rho^{*}$ is as follows [37]:

(6) $$\rho^{*}=\frac{m}{V\times \rho_{s}}$$

where *m* and *V* represent the mass and the volume of the specimens, and $\rho_{s}$ is the density of the Ti6Al4V alloy material in this study, which is 4.45 g/cm^3^.

As shown in Table 6, the relative densities of all samples were within 5% of the design error. The specimens were observed by scanning electron microscopy (SEM) (VEGA3, Texcan, Brno, Czech Republic), and the elemental distribution of the specimens was analyzed by using an energy spectrometer (EDS) (Link-ISIS, Oxford Instruments, China) after sandpapering and polishing in a polishing machine. The phase composition of the molded samples was analyzed by X-ray diffraction (XRD) (DX-2700, Haoyuan, Beijing, China). Internal defects in the specimens were characterized by micro-computed tomography (micro-CT) (X5000, Japan).

## 3. Results

### 3.1. Print Quality

**Figure 4:**
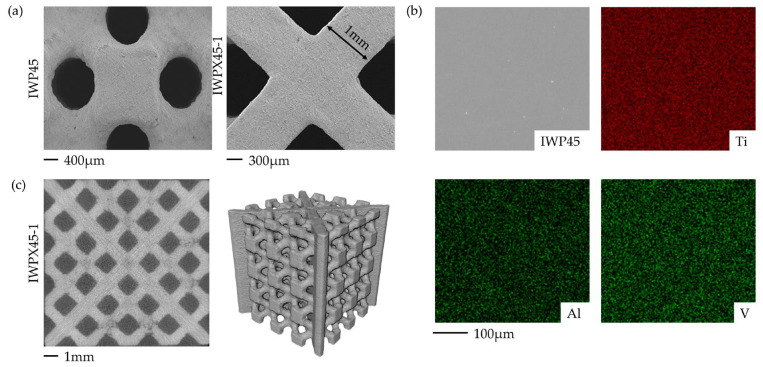
Characterization results of SLM-produced specimens: (**a**) SEM images of the IWP45 and IWPX45-1 specimens; (**b**) EDS element distribution images of specimens; (**c**) micro-CT images of the IWPX45-1 specimen.

Figure 4a shows the SEM images of the specimens, which agree with the CAD model. The surfaces of specimens are slightly rough with traces of laser scanning burnishing and melting. Figure 4b shows the elements in the Ti6Al4V lattice structure detected by EDS. As Figure 4c shows, the plate and strut of the SLM printed sample had no visible fractures or holes, and no visible powder condensation or residue was detected in the inner cavity.

### 3.2. Analysis of Mechanical Properties

**Figure 5:**
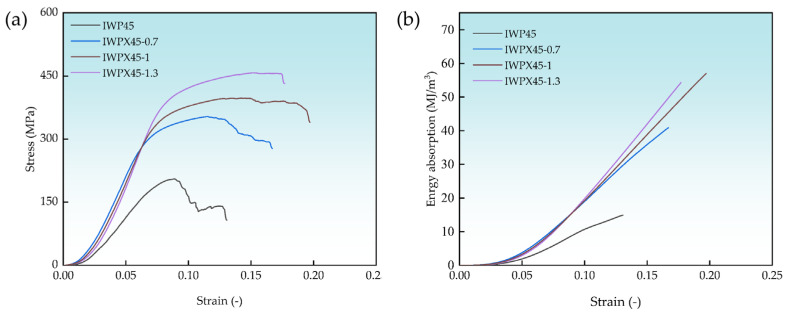
(**a**) Stress–strain curves of lattice structures; (**b**) energy absorption curves of lattice structures.

Figure 5a shows the stress–strain curves for the compression tests of four structures of the same density. Due to the insufficient contact between the SLM printed specimen surfaces and the compression tester indenter surfaces, a brief nonlinear phase occurs before entering the elastic phase [27,38]. After that, the curve enters an elastic phase with a steady increase in the initial stiffness of the structure in full contact with the indenter stress remaining relatively constant with strain. After passing the yield point, the specimen enters the elasto-plastic phase and begins to deform plastically, and as the strain continues, the structure fails and ruptures after the peak stress occurs. The compression results are shown in Table 7.

According to Maxwell’s criterion, the concentration of mass into the plate structure transforms the original bending-based mechanical behavior into the tensile-based mechanical behavior [32] after the addition of the plate, resulting in a substantial strengthening of the axial load-carrying capacity of the IWP structure. The modulus of elasticity of the reinforced structure compared to the same density IWP45 structure increased from 3.81 GPa to 7.43 GPa, the yield strength increased from 178.01 MPa to 359.12 MPa, and the ultimate compressive strength increased from 205.01 MPa to 455.23 MPa. This shows a significant improvement in the mechanical properties of the IWP structure by the plate structure. As shown in Figure 5b, the calculated energy absorption at fracture for the four structures is 14.91 MJ/m^3^, 40.89 MJ/m^3^, 56.96 MJ/m^3^, and 54.28 MJ/m^3^. The energy absorption of IWPX45-1 increased by 282.03% compared to that of IWP45, which indicates that the plate structure significantly improves the energy absorption capacity of the structure in the direction of compression.

**Figure 6:**
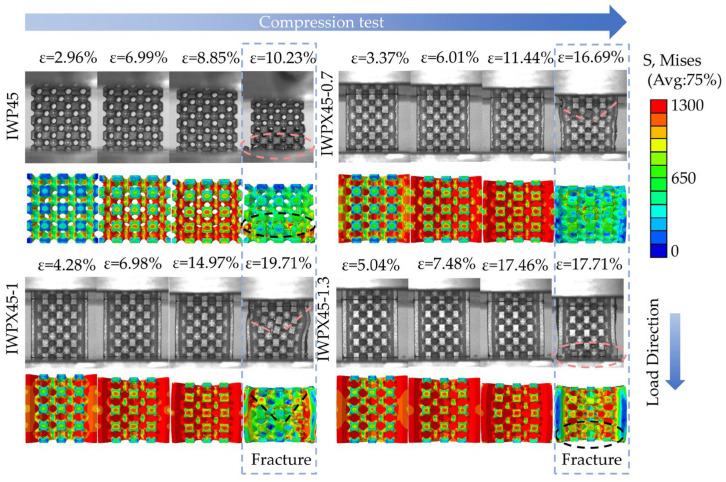
FEA and compression test images of lattice structures.

Figure 6 shows images taken during the compression test and images of the finite element simulation. The stress concentration region on the lattice structure can be seen in the FEA images. The plate structure effectively carries the shear stresses. The simulation tests effectively predicted the fracture location of the lattice structure, which proves the validity of finite element simulation analysis.

The structures deform uniformly before compression begins to 6%. IWP45 reaches the yield point at compressive strains up to 6.99%. At compression to 10.23%, IWP45 starts to collapse layer by layer from the lower layers. This is the same way that IWP structures fracture in the other literature [24]. For the IWPX structure, due to the plate reinforcement, IWPX45-0.7 and IWP45X-1 are gradually deformed by the bending of the plate structure after compression above the ultimate compression stress point. The IWPX45-0.7 structure, which has the smallest plate-to-IWP volume ratio, begins to fracture at compression to 16.69%, showing a V-shaped shear fracture, which is different from IWP45. At compression to 17.71%, the lower layer of IWPX45-1.3 fractured, which was due to an over-representation of the plate structure. At 19.71%, IWPX45-1 fractures with the same pattern as IWPX45-0.7. The result of the compression test suggests that the plate structure can enhance the fracture strain of the IWP structure, and that changing the percentage of the plate structure will change the fracture mode of the structure.

### 3.3. Mechanical Properties of Structures with Different Volume Ratios

**Figure 7:**
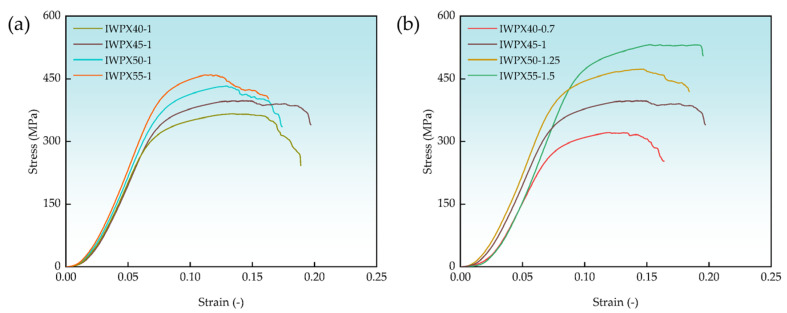
Stress–strain curves of different plate-to-IWP volume ratios: (**a**) stress–strain curves of the same thickness of plate; (**b**) stress–strain curves of the same density of IWP.

The stress–strain curves for the same plate thickness fused with different densities of IWP surface structures are shown in Figure 7a. The plate-to-IWP volume ratio decreases as the density of the IWPX increases. The curves end at the rupture of the axial structure of the sample. As the density of IWPX increases, the compressive strength is enhanced, but after the relative density exceeds 0.45, the fracture strain of IWPX shows a clear tendency to decrease, which is due to the weakening of the deformation resistance of the structure [39].

The stress–strain curves of the same density of the IWP surface structure fused with different thickness plate structures are shown in Figure 7b. The plate-to-IWP volume ratio increases with the increase in density of the IWP-X structure. The difference is that the increase in compressive strength is greater than the decrease in volume ratio with the increase in density of IWP-X, and the trend of decreasing fracture strain is alleviated. This indicates that the strengthening of the plate-to-IWP volume ratio contributes to the improvement of the mechanical properties and plasticity of the overall structure.

**Figure 8:**
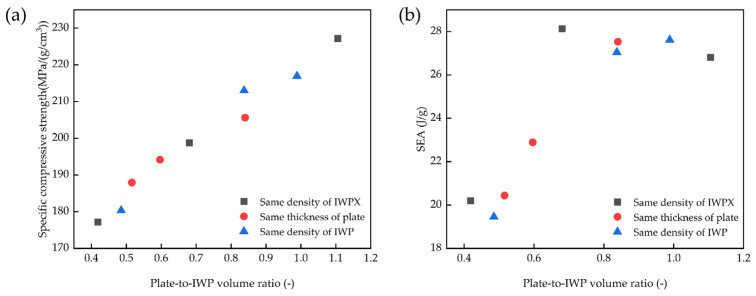
(**a**) Relationship of specific compressive strength versus volume ratio; (**b**) relationship of SEA versus volume ratio.

As shown in Figure 8, the relationship between the volume ratio versus specific compressive strength and SEA is obtained. As the plate volume ratio increases, the structural compressive capacity tends to increase, and the SEA increases rapidly and then decreases slowly. As shown in Figure 8b, the structure with a volume ratio of 0.7-0.8 mm is best in terms of energy absorption.

### 3.4. Comparison with Other Lattice Structures

**Figure 9:**
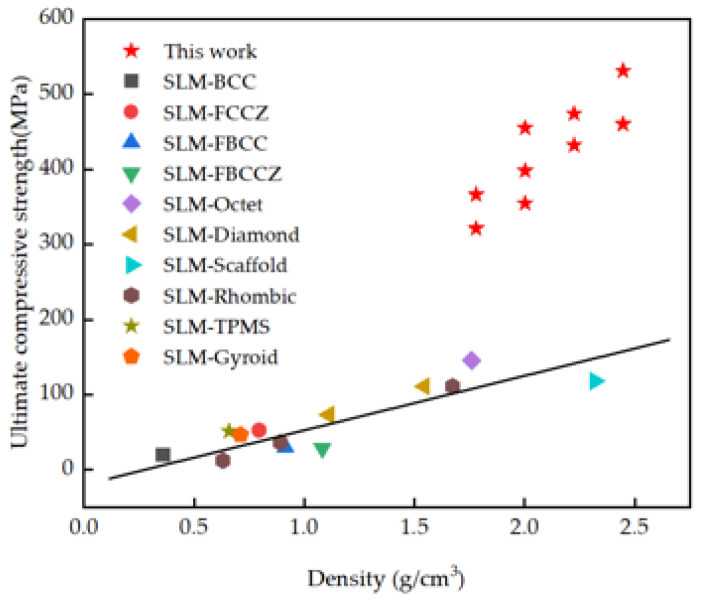
Different lattice structures of the Ti6Al4V alloy prepared by SLM.

The comparison of ultimate compressive strength between IWP-X and other Ti6Al4V lattice structures printed by the SLM [39,40,41,42,43,44] machine is shown in Figure 9, and the red five stars represent the IWP-X structure of this work. The ultimate compressive strength of the IWP structure with the same relative density is higher than the other structures. It shows that the fused X-shaped plate structure has a great improvement in the material properties in the axial direction.

## 4. Conclusions

In this study, an IWPX reinforced structure was first proposed, which fuses an X-shaped plate structure and IWP surface structure. The fusion of the plate structure substantially improves the axial mechanical properties. The mechanical properties and fracture forms of the structure are analyzed by finite element simulation analysis and compression tests on the IWP-X structure with different volume ratios. The following conclusions are mainly drawn:

1. The specific strength of the titanium alloy IWP-X lattice structure reaches 227.22 MPa/(g/cm^3^), which is 1.26 times that of the titanium alloy block (180 MPa/(g/cm^3^)). Compared with the IWP45 structure of the same density, the ultimate compressive strength of IWPX45-1.3 increased by 455.23 MPa (122.06% improvement), and the energy absorption increased by 54.28 MJ/m^3^ (265.03% improvement).
2. The finite element simulation tests effectively predicted the stress distribution and fracture failure site of the structure during the compression test. With the incorporation of the plate structure, the structure transformed from the layer-by-layer fracture of IWP to the V-shaped fracture.
3. As the volume ratio of plate-to-IWP increases, the structural mechanical properties and deformation resistance are enhanced. The SEA reaches its maximum value in the ratio of 0.7 to 0.8. The energy absorption of IWPX45-1 increased by 56.96 MJ/m^3^ (282.03% improvement), which is the largest increase.

This performance-tunable TPMS metamaterial structure effectively enhances the mechanical properties and energy absorption capacity in the load-bearing direction. This provides a new idea for the design of tunable metamaterials for the lightweighting of titanium alloys, which will have important applications in fields such as the aerospace and automotive industry.

## Figures and Tables

**Table 1:** Design parameters of lattice structures.

Structure	Relative Density of I-WP (%)	Thickness of Plate t (mm)	Relative Density (%)	*V_t_*/*V_IWP_*
IWP45	45.00	None	45	None
IWPX45-0.7	37.80	0.7	45	0.42
IWPX45-1	32.60	1	45	0.68
IWPX45-1.3	25.60	1.3	45	1.11
IWPX40-1	26.40	1	40	0.84
IWPX50-1	37.80	1	50	0.60
IWPX55-1	43.00	1	55	0.52
IWPX40-0.7	32.60	0.7	40	0.49
IWPX50-1.25	32.60	1.25	50	0.84
IWPX55-1.5	32.60	1.5	55	1.00

**Table 2:** Parameters of the SLM machine.

Laser Power	Laser Spot Size	Hatch Distance	Scanning Speed	Layer Thickness
280 W	0.1 mm	0.1 mm	1000 mm/s	0.03 mm

**Table 3:** Chemical composition of Ti6Al4V powder.

Element	Ti	Al	V	Fe	O	C	H	N
Mass fraction (%)	Bal	5.5–6.5	3.5–4.5	≤0.25	≤0.2	≤0.08	≤0.012	≤0.05

**Table 4:** Mechanical properties of titanium alloy powder.

Power	Yield Strength (MPa)	Tensile Strength (MPa)	Elongation After Break (%)
Ti6Al4V	1050 ± 50	1230 ± 50	7 ± 3

**Table 5:** The number of nodes and elements.

Structure	Number of Nodes	Number of Elements
IWP45	1,562,279	959,832
IWPX45-0.7	1,649,300	1,019,071
IWPX45-1	1,432,624	874,278
IWPX45-1.3	1,390,934	852,106

**Table 6:** The measurement of structural characterizations.

Structure	Relative Density (%)	Actual Relative Density (%)	Error (%)
IWP45	45.00	46.64 ± 0.52	3.64 ± 1.16
IWPX45-0.7	45.00	46.60 ± 0.36	3.56 ± 0.80
IWPX45-1	45.00	46.85 ± 0.35	4.11 ± 0.78
IWPX45-1.3	45.00	46.82 ± 0.35	4.04 ± 0.78
IWPX40-1	40.00	41.34 ± 0.43	3.35 ± 1.08
IWPX50-1	50.00	51.55 ± 0.85	3.10 ± 1.70
IWPX55-1	55.00	56.45 ± 0.35	2.64 ± 0.63
IWPX40-0.7	40.00	41.13 ± 0.64	2.83 ± 1.60
IWPX50-1.25	50.00	51.95 ± 0.43	3.90 ± 0.86
IWPX55-1.5	55.00	56.55 ± 0.86	2.82 ± 1.56

**Table 7:** The compression results of structures.

Structure	Elastic Modulus (GPa)	Yield Strength (MPa)	Ultimate Compressive Strength (MPa)
IWP45	3.81 ± 0.55	178.01 ± 5.52	205.01 ± 9.12
IWPX45-0.7	6.56 ± 0.68	269.35 ± 7.23	354.79 ± 13.25
IWPX45-1	6.85 ± 0.72	317.77 ± 9.32	398.95 ± 15.82
IWPX45-1.3	7.43 ± 0.85	359.12 ± 11.23	455.23 ± 17.92
IWPX40-1	6.72 ± 0.51	287.25 ± 8.12	366.36 ± 14.52
IWPX50-1	7.20 ± 0.79	339.28 ± 10.52	432.25 ± 16.72
IWPX55-1	7.58 ± 0.88	372.27 ± 11.92	460.65 ± 18.32
IWPX40-0.7	5.86 ± 0.42	250.58 ± 6.82	321.78 ± 11.32
IWPX50-1.25	7.19 ± 0.63	370.24 ± 11.82	474.45 ± 19.12
IWPX55-1.5	7.64 ± 0.32	431.48 ± 11.98	531.36 ± 19.82

## Data Availability

The original contributions presented in this study are included in the article. Further inquiries can be directed to the corresponding author.
